# Inhibitors of Jumonji C domain-containing histone lysine demethylases overcome cisplatin and paclitaxel resistance in non-small cell lung cancer through APC/Cdh1-dependent degradation of CtIP and PAF15

**DOI:** 10.1080/15384047.2021.2020060

**Published:** 2022-01-31

**Authors:** Lei Duan, Ricardo E. Perez, Sarah Calhoun, Carl G. Maki

**Affiliations:** a Department of Cell & Molecular Medicine, Rush University Medical Center, Chicago, IL, USA; b Robert H. Lurie Comprehensive Cancer Center of Northwestern University, Chicago, IL, USA

**Keywords:** Histone demethylase, APC/C, CtIP, PAF15, cisplatin, NSCLC

## Abstract

The Jumonji C domain-containing family of histone lysine demethylases (Jumonji KDMs) have emerged as promising cancer therapy targets. These enzymes remove methyl groups from various histone lysines and, in turn, regulate processes including chromatin compaction, gene transcription, and DNA repair. Small molecule inhibitors of Jumonji KDMs have shown promise in preclinical studies against non-small cell lung cancer (NSCLC) and other cancers. However, how these inhibitors influence cancer therapy responses and/or DNA repair is incompletely understood. In this study, we established cell line and PDX tumor model systems of cisplatin and paclitaxel-resistant NSCLC. We showed that resistant cells and tumors express high levels of Jumonji-KDMs. Knockdown of individual KDMs or treatment with a pan-Jumonji KDM inhibitor sensitized the cells and tumors to cisplatin and paclitaxel and blocked NSCLC in vivo tumor growth. Mechanistically, we found inhibition of Jumonji-KDMs triggers APC/Cdh1-dependent degradation of CtIP and PAF15, two DNA repair proteins that promote repair of cisplatin and paclitaxel-induced DNA lesions. Knockdown of CtIP and PAF15 sensitized resistant cells to cisplatin, indicating their degradation when Jumonji KDMs are inhibited contributes to cisplatin sensitivity. Our results support the idea that Jumonji-KDMs are a targetable barrier to effective therapy responses in NSCLC. Inhibition of Jumonji KDMs increases therapy (cisplatin/paclitaxel) sensitivity in NSCLC cells, at least in part, by promoting APC/Cdh1-dependent degradation of CtIP and PAF15.

## Introduction

Non-small cell lung cancer (NSCLC) accounts for the vast majority (80–85%) of all cases. Standard NSCLC treatment includes aggressive platinum and taxane-based chemotherapy. However, most NSCLC tumors respond poorly to treatment. Further, patients that respond to initial treatments inevitably develop recurrent, therapy-resistant tumors and die from their disease. To improve outcomes in NSCLC patients, it is essential to identify the barriers to effective therapy responses and ways to overcome them.

In recent years, Jumonji-domain histone lysine demethylases (Jumonji-KDMs) have emerged as key players in NSCLC and other cancers and potential therapeutic targets. These enzymes remove methyl groups from histone H3 lysines 4, 9, 27, or 36 (H3K4, H3K9, H3K27, or H3K36) and regulate processes such as chromatin compaction, gene transcription, and DNA repair.^1–5^ High expression of Jumonji-KDMs has been reported across multiple cancer types including breast, prostate, colon, and NSCLC.^6,7^ In several cases, this high expression is associated with worse patient outcome, including in NSCLC.^8–11^ Moreover, multiple studies including our own have shown high expression and activity of Jumonji-KDMs promotes resistance to chemotherapy and radiation.^8,12–15^ These findings support targeting Jumonji KDMs to improve therapy responses and outcomes inNSCLC and other cancers. Accordingly, multiple inhibitors of Jumonji-KDMs are in preclinical development. However, over 30 Jumonji-KDMs have been identified, many with overlapping functions.^2^ Thus, inhibiting single or small groups of Jumonji KDMs may have limited impact. Pan-inhibitors that target most or all Jumonji KDMs may have the greatest therapeutic benefit.

In this study, we established cell line and PDX tumor model systems of cisplatin and paclitaxel-resistant NSCLC. We showed that resistant cells and tumors express high levels of Jumonji-KDMs. Knockdown of individual KDMs or treatment with a pan-Jumonji KDM inhibitor sensitized the cells and tumors to cisplatin and paclitaxel and blocked NSCLC in vivo tumor growth. Mechanistically, we found inhibition of Jumonji-KDMs triggers APC/Cdh1-dependent degradation of CtIP and PAF15, two DNA repair proteins that promote repair of cisplatin and paclitaxel-induced DNA lesions. Knockdown of CtIP and PAF15 sensitized resistant cells to cisplatin, supporting that their degradation when Jumonji KDMs are inhibited contributes to cisplatin sensitivity. Our results support the idea that Jumonji-KDMs are a targetable barrier to effective therapy responses in NSCLC. Inhibition of Jumonji KDMs increases therapy (cisplatin/paclitaxel) sensitivity in NSCLC cells, at least in part, by promoting APC/Cdh1-dependent degradation of CtIP and PAF15.

## Results

### Increased expression of Jumonji KDMs promotes cisplatin and paclitaxel resistance in NSCLC cells and xenograft tumors

We wished to examine the relationship between Jumonji KDMs and therapy resistance in NSCLC. To this end, we first isolated cisplatin (CP)-resistant populations from three different NSCLC cell lines after repeated exposure to CP. We compared mRNA expression of different Jumonji KDMs in the parental and CP-resistant (CPR) cells. Notably, some Jumonji KDMs were increased in response to CP treatment (Fig S1A). This is consistent with previous studies showing expression of different Jumonji KDMs induced in response to CP.^16,17^ CPR cells expressed elevated levels of multiple Jumonji KDMs compared to parental controls, both basally and in response to CP. Some of these KDMs (KDM3A, KDM4B, KDM5A, and KDM6A) were commonly overexpressed in all 3 CPR cell populations (names in red in Fig. S1A). We isolated paclitaxel (PTX) resistant NSCLC cells using a similar approach. The PTX-resistant (PTXR) cells also showed high expression of various Jumonji KDMs including KDM4B, KDM5A, and KDM6A (Fig. S1B).

**Figure 1.**
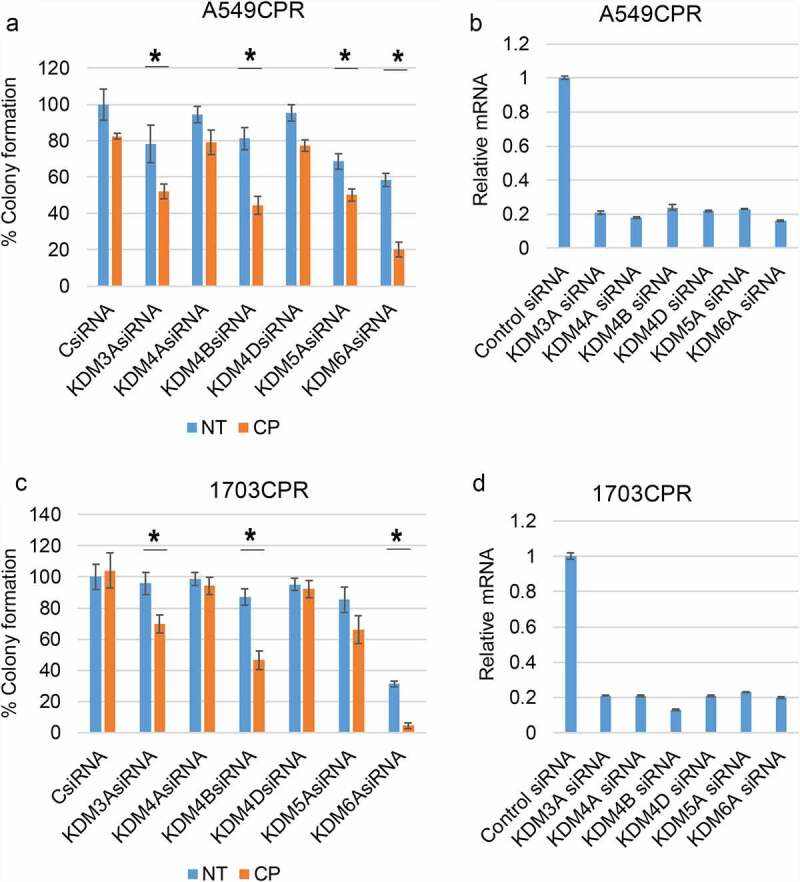
Knockdown of KDMs sensitizes resistant cells to CP. (a) and (c). CP-resistant A549 and 1703 cells were transfected with control siRNA (CsiRNA) or the indicated KDM siRNA and then treated with treated with vehicle (NT) or CP (1 µM) for 48 h. After drug removal, the cells were allowed to recover for 10 days. % colony formation is presented as graphs with SD indicated. There are significant differences (indicated with single asterisks above bars, p ˂0.05) between vehicle and CP treated conditions in some KDM siRNA transfected cells. There are significant differences in CP-treated A549 CPR cells between control siRNA and KDM3A siRNA (*P* = .002), KDM4B siRNA (*P* = .002), KDM5A siRNA (*P* = .018), and KDM6A siRNA (*P* = .000). There are significant differences in CP-treated 1703 CPR cells between control siRNA and KDM3A siRNA (*P* = .046), KDM4B siRNA (*P* = .05), KDM5A siRNA (*P* = .05), and KDM6A siRNA (*P* = .002) in A549. (b) and (d). qPCR analysis shows knockdown of the individual genes.

**Figure 2.**
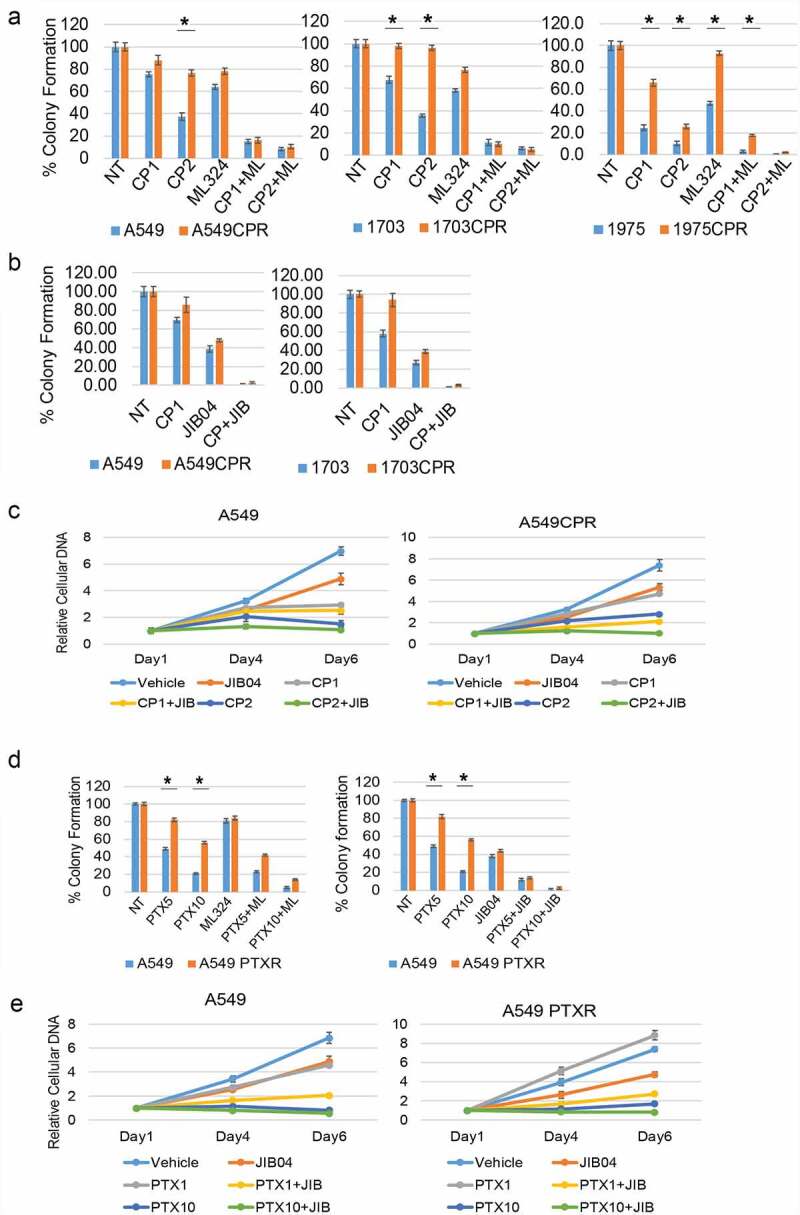
Inhibition of KDMs sensitizes resistant cells to CP and PTX. (a) Parental and resistant cells were treated with vehicle (NT) or CP (1 µM or 2 µM) with or without ML324 (10 µM) for 48 h. (b) Parental and resistant cells were treated with vehicle (NT) or CP (1 µM) with or without JIB04 (1 µM) for 48 h. Colony formation was determined 10d after drug removal. Triplicate results are shown. There are significant differences in CP treated conditions between parental and CPR cell lines (indicated with single asterisks above bars, p ˂0.05). There are significant differences between CP1 and CP1+ ML324 conditions in A549 (*P* = .000), ACPR (*P* = .000), 1703 (*P* = .000), 1703CPR (*P* = .012), 1975 (*P* = .033), and 1975CPR (*P* = .039) cells and between CP2 and CP2+ ML324 conditions in A549 (*P* = .035), ACPR (*P* = .000), 1703 (*P* = .021), 1703CPR (*P* = .000), 1975 (*P* = .045), 1975CPR (*P* = .027) cells. There are significant differences between JIB04 and CP1+ JIB04 conditions in A549 (*P* = .000), ACPR (*P* = .000), 1703 (*P* = .001), and 1703CPR (*P* = .002) cells. (c) A549 and A549CPR cells were treated with vehicle, CP (1 µM or 2 µM) with or without JIB04 (1 µM) for two days and analyzed for cell proliferation for 6 days with staining of cellular DNA. Average (quadruplicate) relative DNA is presented with SD indicated. There is a significant difference between CP1 treated A549 and ACPR cells (*P* = .038). There is a significant difference between CP1 and CP1+ JIB04 treated ACPR cells (*P* = .043). (d) Parental and resistant cells were treated with vehicle (NT) or PTX (5 nM or 10 nM)) with or without ML324 (10 µM) or JIB04 (1 µM) for 48 h. Colony formation was determined 10d after drug removal. Triplicate results are shown. There are significant differences between A549 and A549PTXR cells treated with PTX (indicated above bars). There are significant differences between PTX5 and PTX5+ ML324 in A549 (*P* = .041) and APTXR (*P* = .021) cells and between PTX10 and PTX10+ ML324 in A549 (*P* = .036) and APTXR (*P* = .028) cells. (e) A549 and A549PTXR cells were treated with vehicle, PTX (1 nM or 10 nM) with or without JIB04 (1 µM) for two days and analyzed for cell proliferation for 6 days with staining of cellular DNA. Average (quadruplicate) relative DNA is presented with SD indicated. There is a significant difference between PTX1 treated A549 and ACPR cells (*P* = .022). There is a significant difference between JIB04 and PTX1+ JIB04 treated A549 cells (*P* = .031) and ACPR cells (*P* = .046).

Two approaches were taken to test if the Jumonji KDMs contribute to CP and PTX resistance in these cells. First, we knocked down individual KDMs in CPR cells and measured their sensitivity to CP by colony forming ability. As shown in Figure 1, knockdown of KDM4B and KDM6A caused a pronounced sensitization to CP in A549CPR and 1703 CPR cells. KDM6A knockdown alone also reduced survival, especially in 1703CPR cells. KDM5A and KDM3A knockdown increased CP sensitivity in the resistant cells though to a lesser extent than KDM4B/6A knockdown, while KDM4A and 4D knockdown caused no sensitization. Next, we treated parental and resistant cells with two different Jumonji KDM inhibitors; ML324 (inhibits KDM4 family members) or JIB04 (pan-inhibitor of all Jumonji KDMs).^18^ We then monitored survival of the cells in response to CP and PTX treatment by colony forming ability. As shown in Figure 2, ML324 and JIB04 caused a striking sensitization of both parental and the resistant cells to both cisplatin and paclitaxel (Figure 2a, b, d). Notably, there are no significant differences between parental and resistant cells in the CP+ML324 and CP+JIB04 conditions except between 1975 and 1975 CPR treated with CP+ML324, suggesting ML324 and JIB04 had a greater effect in sensitizing resistant cells to CP. We also compared proliferation in parental and resistant cells treated with CP or PTX alone or in combination with Jumonji KDM inhibitor (Figure 2c and e). The CPR and PTXR cells were better able to maintain proliferation than the parental cells in response to CP and PTX treatment, especially at the lower (1 µM) doses. JIB04 cotreatment with CP/PTX blocked proliferation in both parental and resistant cells. In total, the results demonstrate that Jumonji KDMs contribute to CP and PTX resistance in NSCLC cells.

**Figure 3.**
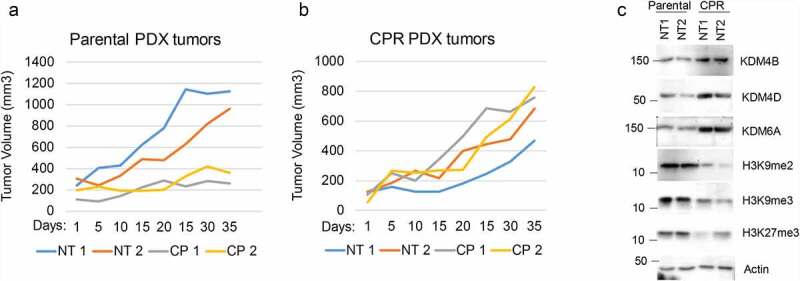
CP-resistant PDX tumors express higher KDMs. (a) Two mice bearing parental PDX tumors were treated with vehicle or CP (5 mg/kg) once and tumor growth was monitored. (b) PDX tumors were treated with five rounds of CP until the tumors became CP resistant. Two mice bearing CP-resistant PDX tumors were treated with vehicle or CP (5 mg/kg) once and tumor growth was monitored. (c) Expression of the indicated proteins in parental and CP-resistant PDX tumors were analyzed with immunoblot of tumor lysates.

**Figure 4.**
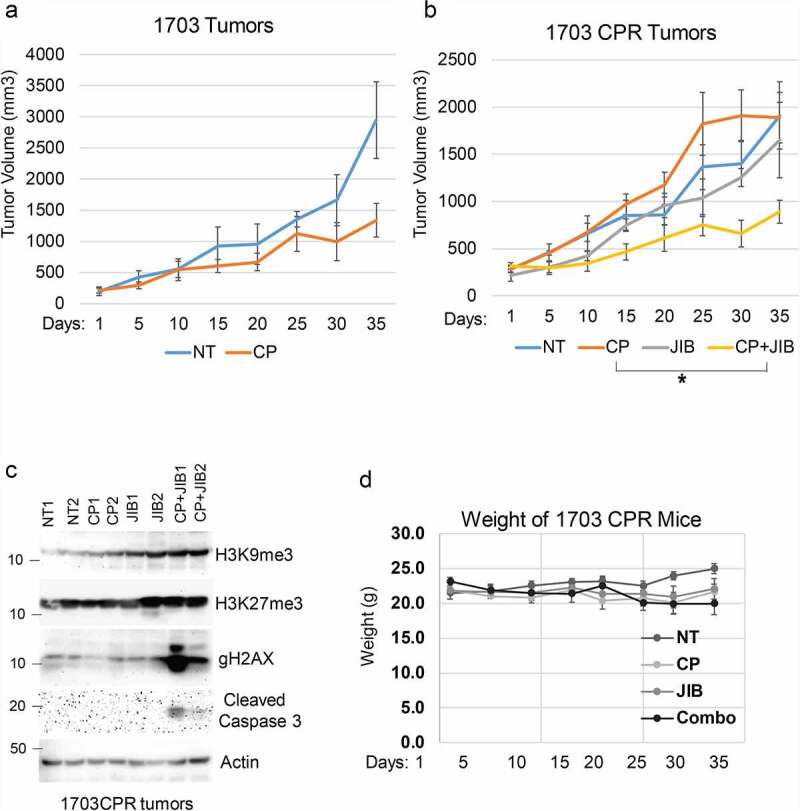
Combination JIB04 plus CP overcome CP-resistance in vivo. (a),(b) Mice bearing 1703 or 1703CPR tumors treated with vehicle (NT), CP (5 mg/kg, once), JIB04 (50 mg/kg/day, 5 days/week), or CP plus JIB04 and tumor growth monitored. There is a significant difference between vehicle and CP-treated 1703 tumors (*P* = .042). There is no significant difference between vehicle and CP (*P* = .25) and vehicle and JIB04 (*P* = .99) in 1703CPR tumors. There is a significant difference between vehicle and CP plus JIB04 (*P* = .003) treated 1703CPR tumors. There is a significant difference between CP plus JIB04 and CP (*P* = .043) or JIB04 (*P* = .049). (c) Tumor lysates (two tumors/group) were immunoblotted for the indicated proteins. (d) Average body weights of the mice bearing 1703CPR tumors are presented ± SE.

We next wished to ask if Jumonji KDMs are overexpressed in therapy resistant NSCLC tumors and can be targeted in an in vivo setting. To this end, we first established a NSCLC PDX tumor in mice and then treated the mice repeatedly with CP until the tumor became non-responsive (Figure 3a and b). We then determined levels of different Jumonji KDMs as well as levels of methylated histones in tumor protein lysates from parental PDX tumors and the resistant (CPR) PDX tumors that were no longer responsive to CP. As shown in Figure 3c, the CP-resistant PDX tumors showed high expression of different Jumonji KDMs (KDM4B, 4D, 6A) and reduced histone methylations (H3K9me2/me3 and H3K27me3). KDM3A and KDM5A expression were similar in CP-resistant tumors compared with parental tumors (data not shown). The results demonstrate that CP-resistant NSCLC tumors show high expression and activity of different Jumonji KDMs. Lastly, we asked if Jumonji KDM inhibitors could overcome therapy resistance in vivo, in this case using matched 1703 and 1703CPR cells. 1703 and 1703CPR cells were grown as xenograft tumors in mice. The tumor-bearing mice were then treated with either vehicle, CP, JIB04, or CP+JIB04 and tumor growth monitored. As shown in Figure 4a and b, parental 1703 xenograft tumor growth was inhibited by CP treatment while 1703CPR xenograft tumor growth was largely unaffected by either CP or JIB04 alone. However, combined treatment with CP+JIB04 caused a pronounced reduction in growth of 1703CPR tumors (Figure 4b) while having minimal effect on body weight (Figure 4d), supporting the idea that Jumonji KDM inhibitors can be used to sensitize resistant tumors to CP in vivo. Combined treatment with CP+JIB04 was associated with increased DNA damage (evidenced by increased gH2AX), increased apoptosis (evidenced by cleaved caspase 3), and increased histone methylation in tumor protein lysates (Figure 4c).

### Jumonji KDM inhibitors cause depletion of CtIP and PAF15 DNA repair proteins

**Figure 5.**
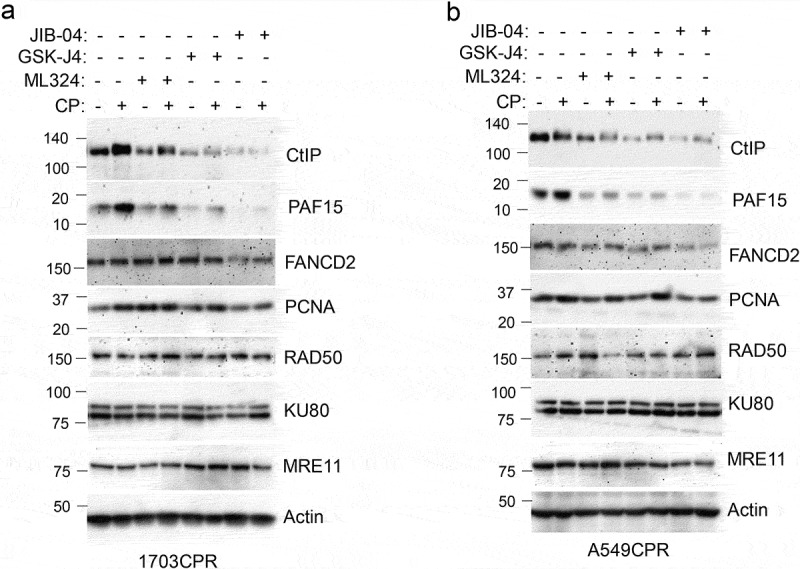
Jumonji KDM inhibitors reduce CtIP and PAF15 protein levels. 1703CPR and A549CPR cells were treated with CP (10 µM) and/or ML324 (10 µM), GSK-J4 (10 µM), or JIB04 (2 µM) for 24 hrs. Lysates were blotted for the indicated proteins.

**Figure 6.**
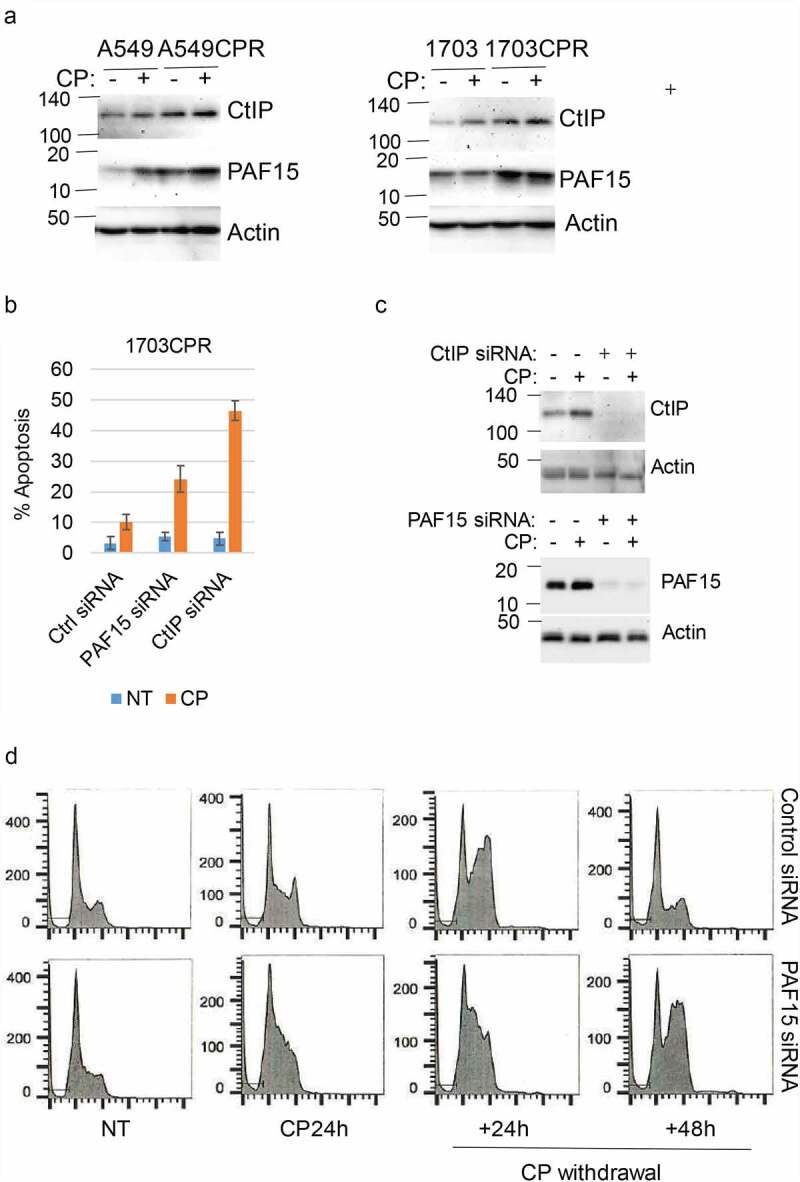
Knockdown of CtIP and PAF15 sensitizes cells to CP. (a) A549/A549CPR and 1703/a703CR cells were treated with vehicle or CP (10 µM) for 24 h. Lysates were immunoblotted for the indicated proteins. (b) 1703CPR cells were transfected with control siRNA (ctrl siRNA) or PAF15 or CtIP siRNA and then treated with vehicle or CP (10 µM) for 72 h. Apoptosis was assessed by determining the percentage of cells with sub-G1 DNA content by flow cytometry. Average (triplicate) % Sub-G1 cells (apoptosis) were presented with SD indicated. There are significant differences between control siRNA and PAF15 siRNA (*P* = .041) and between control siRNA and CtIP siRNA (*P* = .000) in CP treated conditions. (c) Lysates were immunoblotted for the indicated proteins. (d) 1703CPR cells were transfected with control siRNA or PAF15 siRNA and then treated with CP (5 µM) for 24 h. The cells were then grown in drug free media for another 48 h. Cells were harvested at the indicated time points and analyzed for cell cycle. Cell cycle histograms are presented.

**Figure 7.**
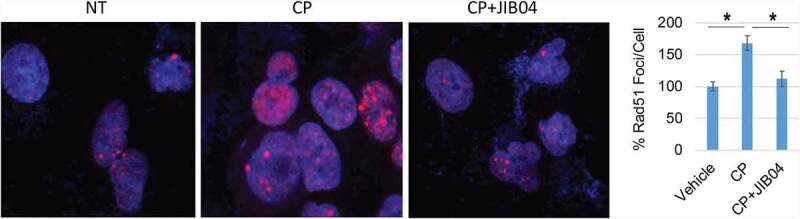
JIB04 reduces Rad51 Foci in CP-treated cells. 1703 CPR cells were treated with vehicle, CP, or CP plus JIB04 for 24 hrs. Cells were immunostained for Rad51. Rad51 foci were counted (100 cells/slide) and average (triplicate) % foci relative to the control are presented with SD indicated. There are significant differences between vehicle/CP and CP/CP+JIB04 conditions (*P* values indicated).

DNA repair is a major mechanism for therapy resistance and poor outcome in NSCLC.^19^ CP and other platinum agents induce DNA crosslinks and subsequent double strand breaks (DSBs). Multiple pathways are implicated in the repair of platinum-induced DNA lesions, including homologous repair (HR), non-homologous end joining (NHEJ), nucleotide excision repair (NER), translesion synthesis (TLS), and the Fanconi Anemia (FA) pathway.^20,21^ While PTX targets microtubules, it can also induce DSBs that are repaired through HR.^22^ We examined expression of multiple DNA repair proteins in NSCLC cells treated with CP and/or Jumonji KDM inhibitors. DNA repair proteins examined and their associated repair pathway are: CtIP (HR), PAF15 (TLS), FANCD2 (FA), PCNA (NER, TLS), RAD50 (HR, NHEJ), MRE11 (HR, NHEJ). Jumonji KDM inhibitors used were ML324 (inhibits KDM4 proteins), GSK-J4 (inhibits KDM6s), and JIB04 (inhibits all Jumonji KDMs). As shown in Figure 5, expression of most repair proteins was unaffected by the Jumonji KDM inhibitors. However, both CtIP and PAF15 were strikingly reduced by JIB04 alone or CP+JIB04, and partially reduced by ML324 and GSK-J4 alone or with CP. We also found both CtIP and PAF15 are expressed at higher levels in CP resistant cells compared to their parental cells (Figure 6a). Altogether, these results suggest that increased CtIP and PAF15 expression are involved in acquired resistance to CP and that inhibition of Jumonji KDMs may sensitize cells to therapy, in part, by depleting CtIP and PAF15 and inhibiting DNA repair. To examine this possibility directly, we siRNA-depleted CtIP and PAF15 in 1703CPR cells and then monitored sensitivity of these cells to CP-induced apoptosis. Apoptosis was assessed by determining the percentage of cells with sub-G1 DNA content by flow cytometry. As shown in Figure 6b and c, depletion of CtIP and PAF15 sensitized the cells to CP-induced apoptosis. This supports the idea that depletion of CtIP and PAF15 in JIB04 treated cells contributes to CP sensitivity. We also analyzed cell cycle profiles in control cells and PAF15-depleted cells after treatment and removal of CP. Control cells were accumulated in S and G2/M phases 24 h after CP removal, but progressed through the cell cycle and resumed a normal cell cycle distribution 48 h after CP removal. In contrast, the PAF15 depleted cells remained accumulated in S and G2/M phase at both time points after CP removal, suggesting PAF15-depleted cells have increased replication fork stalling that could contribute to their killing in CP-treated cells (Figure 6d). Lastly, we determined the effect of Jumonji KDM inhibition on RAD51 nuclear foci, which is a surrogate marker of HR repair. As shown in Figure 7, JIB04 blocked the CP-induced increase in RAD51 nuclear foci, indicating JIB04 reduces HR repair.

### Degradation of CtIP and PAF15 in response to Jumonji KDM inhibition is APC/Cdh1 dependent

**Figure 8.**
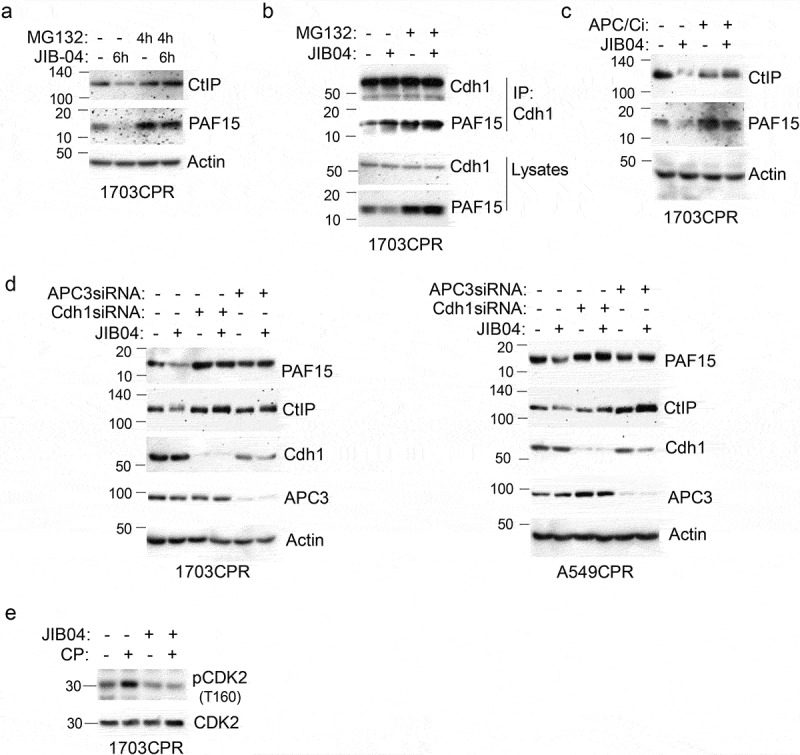
JIB04 induces APC/Cdh1-dependent degradation of CtIP and PAF15. a. 1703 CPR cells were treated with JIB04 (2 µM) for 6 h ± MG132 (10 µM) for 4 h and blotted. b. Lysates were IP’ed with Cdh1 antibodies and blotted for the indicated proteins. c. Cells were treated with JIB04 ± TAME (APC/Ci, 10 µM) for 6 h. d. Cells transfected with APC3 or Cdh1 siRNA were treated with JIB04 for 6 h and blotted. e. Cells were treated with CP and/or JIB04 for 24 h. Lysates were immunoblotted for the indicated proteins.

We sought the mechanism how Jumonji KDM inhibition promotes CtIP and PAF15 degradation. Previous reports showed CtIP and PAF15 are degraded by the CDH1-associated anaphase-promoting complex (APC/CCdh1).^23,24^ APC/C is a large complex that includes a cullin (Apc2) and RING (Apc11) subunit. Activity of APC/C is controlled by activating subunits Cdh1 and Cdc20.^25^ Binding of Cdh1 to its substrates such as PAF15 and CtIP activates APC/C to degrade these proteins. We used several approaches to ask if reduced CtIP and PAF15 levels in JIB04 treated cells results from APC/^Cdh1^ -mediated degradation. First, CtIP and PAF15 levels were determined in 1703CPR cells that were treated with JIB04 for 6 h and the proteasome inhibitor MG132 for the last 4 h. The results showed MG132 blocked the reduction in PAF15 and CtIP caused by JIB04 (Figure 8a), confirming the reduction results from proteosomal degradation. We also found PAF15 coimmunoprecipitated with Cdh1 in JIB04-treated cells and that this was increased by MG132 (Figure 8b). Second, CtIP and PAF15 levels were determined in 1703CPR cells treated with JIB04 and/or the APC/C inhibitor TAME (APC/Ci). The results showed the reduction in PAF15 and CtIP caused by JIB04 was blocked by TAME (Figure 8c), supporting that the reduction is APC/C dependent. Third, Cdh1 and APC3 were siRNA depleted from 1703CPR and A549CPR cells and the effect of JIB04 on CtIP and PAF15 levels tested. The reduction in CtIP and PAF15 by JIB04 was rescued by knock-down of Cdh1 or APC3 (Figure 8d). APC/C is suppressed by CDK2-mediated phosphorylation of CDH1, and CP is reported to activate CDK2.^26,27^ We found that CP induced activation of CDK2 (indicated by phosphorylation at T160) is blocked by cotreatment with JIB04 (Figure 8e) suggesting inhibition of KDMs blocks CDK2 activation in CP-treated cells. Reduced CDK2 activation may contribute to activation of APC/C in these cells. In total, the results indicate that JIB04 induces APC/Cdh1 -dependent degradation of CtIP and PAF15.

## Discussion

In the current study, we established cell and tumor models to examine if and how Jumonji KDMs contribute to therapy resistance in NSCLC. We found CP and PTX-resistant NSCLC cell lines and a CP-resistant NSCLC tumor selected in vivo express high levels of different Jumonji KDMs, including KDM4 and KDM6 family members. Knockdown of individual KDMs or treatment with KDM inhibitors sensitized resistant cell lines and tumors to CP and PTX. These findings are consistent with previous studies and support the idea that high expression of Jumonji KDMs promotes therapy resistance in NSCLC. Notably, some Jumonji KDMs were expressed at lower levels in CP/PTX-resistant cell lines compared to their parental counterparts (Fig S1). This raises the possibility that reduced expression of certain Jumonji KDMs might also contribute to therapy resistance in individual cell lines.

Previous studies reported that Jumonji KDM inhibitors could sensitize cells to radiation by increasing histone methylation and blocking recruitment of repair proteins to DNA damage sites.^1^ Our studies showed Jumonji KDM inhibitors caused a decrease in Chk1 protein stability and ATR activation important for DNA damage response.^12^ The current report presents an additional, new mechanism for therapy sensitization by Jumonji KDM inhibition that involves induced degradation of DNA repair proteins via the APC/Cdh1 ubiquitin-ligase complex. Specifically, we found the Jumonji KDM inhibitors ML324, GSKJ4, and JIB04 caused degradation of two DNA repair proteins: CtIP, which functions in HR repair, and PAF15, which functions in TLS repair. The proteasome inhibitor MG132 and the APC/C inhibitor TAME blocked depletion of CtIP and PAF15 in JIB04-treated cells, confirming that the decrease is due to proteasome and APC/C-dependent degradation. Knockdown of Cdh1 and APC3 also blocked the decrease in CtIP and PAF15 upon JIB04 treatment, indicating the decrease is APC3 and Cdh1 dependent. Lastly, knockdown of CtIP and PAF15 sensitized resistant cells to CP-induced apoptosis. The results indicate JIB04 sensitizes NSCLC cells to CP, at least in part, by promoting APC/Cdh1-dependent degradation of CtIP and PAF15.

A question that arises from our studies is how APC/Cdh1 is activated by Jumonji KDM inhibition. The activity of APC/C is controlled in part by the cyclin-dependent kinase CDK2. Thus, active CDK2 can phosphorylate Cdh1 to inhibit APC/C in G1 and S-phase.^27–29^ Therefore, we speculated Jumonji KDM inhibition could activate APC/Cdh1 by inhibiting CDK2. To explore this, we determined levels of activated CDK2 in cells treated with CP and JIB04 using an antibody that detects the activating phosphorylation of CDK2 at T160. CP is reported to activate CDK2^26^ and, consistent with this, we found levels of CDK2 phosphorylated at T160 (activated) increased in CP-treated 1703CPR cells. However, cotreatment with JIB04 blocks the increase in CDK2 T160 phosphorylatioin in CP-treated cells (Figure 8e). Thus, Jumonji KDM inhibition blocks CDK2 activation by CP that may contribute to activation of APC/C.

A second question is how CtIP and PAF15 degradation sensitizes NSCLC cells to CP. CtIP is a 5ʹ to 3ʹ endonuclease that binds the MRN complex and facilitates strand invasion during HR.^30^ CtIP is an APC/Cdh1 substrate and we found CtIP is degraded in response to JIB04. HR is important for repair of CP and PTX-induced DNA double strand breaks. CtIP depletion sensitized resistant cells to CP, and JIB04 reduced HR evidenced by reduced RAD51 foci formation in CP-treated cells. In our ongoing studies, we found co-treatment with the HR inhibitor RI-1 sensitized A549CPR and 1703CPR cells to CP, confirming that blocking HR can overcome CP resistance (Fig. S2). The most likely scenario is that CtIP depletion/degradation sensitizes cells to CP by inhibiting HR. The role of PAF15 (PCNA associated factor 15) is less clear. PAF15 is believed to function in TLS repair important for DNA replication past CP-induced DNA crosslinks.^31^ During TLS, the normal stalled DNA polymerase is removed and replaced with a TLS polymerase that progresses past the crosslink. The TLS pol is then replaced with normal polymerase that continues normal DNA replication (this is referred to as fork restart). In a previous study, Povlsen et al. showed that PAF15 depletion slowed replication fork velocity, sensitized cells to CP, and prevented removal of TLS pol from UV damage sites.^31^ Thus, PAF15 depletion could sensitize cells to CP by affecting TLS repair in this way. Our cycle analysis showed that in PAF15 knockdown cells cell cycle recovery and progress was blocked in S phase after removal of CP, which is consistent with Povlsen et al.’s finding that PAF15 is required for TLS and fork restoration in CP-treated cells. Altogether, our findings suggest that KDM inhibitors can activate APC/C to degrade CtIP and PAF15, leading to impaired HR and fork restoration in CP-treated cells that contribute to cell death.

In sum, we found high expression of Jumonji KDMs contributes to CP and PTX resistance in NSCLC cell lines and tumors. Inhibiting Jumonji KDMs overcomes this resistance in part by triggering APC/Cdh1-dependent degradation of CtIP and PAF15.

## Materials and methods

### Cells and reagents

A549, H1703, and H1975 cells were from ATCC. All cell lines were grown in RPMI medium with 10% fetal bovine serum (FBS), penicillin (100 U/mL) and streptomycin (100 µg/mL). Cells were plated 48 h before treatment. Cisplatin was obtained from Sigma Aldrich (St. Louis, MO, USA). ML324, JIB04, GSK-J4 and Paclitaxel were from Selleck Chemicals (Houston, TX, USA). TAME is from Cayman Chemical (Ann Arbor, MI, USA).

### Immunoblotting

Whole cell extracts were prepared by scraping cells in RIPA buffer, resolved by sodium dodecyl sulfate polyacrylamide gel electrophoresis (SDS-PAGE) and transferred to polyvinylidene difluoride membranes (Thermo Fisher Scientific, Waltham, MA, USA). Antibodies to H3K9me2 (D85B4), H3K9me3 (D4W1U), H3K27me3 (C36B11), H3K36me3 (D5A7), H3 (D1H2), JMJD2A (KDM4A) (C70G6), JMJD2B (KDM4B) (D7E6), phospho-CDK2 (T160), CDK2, CtIP, PAF15, PCNA, γH2AX (20E3) were from Cell Signaling (Boston, MA, USA); β-actin (C4) and FANCD2 antibodies were from Santa Cruz (CA, USA). UTX (KDM6A) (A302-374A) antibodies were from Bethyl laboratories (Montgomery, TX). JMJD2D (KDM4D) antibodies (NBP1-03357APC) were from Novus Biologicals (Centennial CO, USA). RAD50 and RAD51 antibodies were from Abcam (Cambridge, MA, USA). MRE11 and KU80 antibodies were from Elabscience (Houston, TX, USA). Primary antibodies were detected with goat anti-mouse or goat anti-rabbit secondary antibodies conjugated to horseradish peroxidase were from Invitrogen using Immobilon Western HRP Substrate from EMD Millipore (Burlington, MA). Experiments were conducted three times with representative one presented.

### Flow cytometry

For cell cycle analysis, cells were harvested and fixed in 25% ethanol overnight. The cells were then stained with propidium iodide (25 µg/ml, Calbiochem). Flow cytometry analysis was performed on a Gallios™ Flow Cytometer (Beckman Coulter). Orange emission from propidium iodide (PI) was filtered through a 585/42 nm bandpass filter using linear amplification. A minimum of 10,000 events were collected on each sample. Cell cycle analysis of DNA histograms was performed with FlowJo 10 software (BD Biosciences). Cell population was gated manually into Sub-G1, G1, S, and G2/M phases. Sub-G1 cells were taken as apoptotic cells. Experiments are conducted in triplicate and repeated at least one more time. Average value from one representative experiment is presented with SD indicated as error bars.

### Colony formation assay

Cells were plated in 6-well plates with 200 cells/well in triplicate for 24 hrs. Cells were then treated with drugs for 24 hrs and then released of drugs. Cell were allowed to recover for 3 weeks to form colonies. Colonies were stained with 1% methylene blue (Sigma) in ethanol and number of positive colonies was counted. Experiments are conducted in triplicate and repeated at least one more time. Average value from one representative experiment is presented with SD indicated as error bars. Single asterisks were used on plots to display the difference between datasets to indicate statistically significant differences (*p* values less than 0.05).

### siRNA-mediated transient knockdown

KDM3A/4A/4B/4D/5A/6A siRNA, PAF15 siRNA, CtIP siRNA (On-target plus smart pool), and Control siRNA (On-target plus siControl non-targeting pool) were purchased from GE Dharmacon (Lafayette, CO) and were transfected according to the manufacturer’s guidelines using DharmaFECT I reagent.

### RNA isolation and real-time quantitative PCR analysis

Total RNA was prepared using Total RNA Mini Kit (IBI Scientific, IA, USA); the first cDNA strand was synthesized using High Capacity cDNA Reverse Transcription Kit (Applied Biosystems, CA, USA). Manufacturers’ protocols were followed in each case. PCR primers for histone demethylases are listed in Table S1. **SYBR** green PCR kit (**Midwest Scientific, St. Louis**, USA) was used according to the manufacturer’s instructions. QuantStudio™ 6 was used as follows: activation at 95°C; 2 minutes, 40 cycles of denaturation at 95°C; 15 seconds and annealing/extension at 60°C; 60 seconds, followed by melt analysis ramping from 60°C to 95°C. Relative gene expression was determined by the ΔΔC_t_ method using β-Actin to normalize. Experiments are conducted in triplicate and repeated at least one more time. Average value from one representative experiment is presented with SD indicated as error bars.

### Proliferation assay with Hoechst staining of cellular DNA

Cells were plated in 96-well plates (2 K cells/well) in quadruplicate and treated with vehicle or the indicated drugs. The cells were harvested at day 1, day 4, and day 6 in PBS buffer. The cells then underwent three rounds of standard freeze and thaw followed staining with Hoechst 33342 (10 µM in PBS buffer). The fluorescence intensity of Hoechst 33342 was determined using a BioTek Mx microplate reader with an excitation of 361 nm and an emission of 497 nm. Average relative fluorescence intensity (day 1 vehicle-treated conditions are set as 1) of each treatment condition was presented as graphs with SD indicated.

### Confocal immunofluorescence microscopy

For immunofluorescence analysis, cells were cultured on glass coverslips, fixed in 4% formaldehyde/PBS, permeabilized with 0.5% Triton X-100 for 5 min, and stained with anti-RAD51 antibodies followed by Alexa Fluor 564-conjugated secondary Abs. The stained cells were mounted in mounting medium with Dapi (Life Technologies), and images were acquired with a confocal microscope (Zeiss LSM 700) under X200 or X400 magnifications.

### In vivo xenografting and therapy

NOD.Cg-*Prkdc^scid^*/J (NOD scid) and NOD.Cg-*Prkdc^scid^Il2rg^tmlWjl^*/Sz (NOD-SCID IL2rγ^null^; NSG) mice were obtained from the Jackson Laboratory (Bar Harbor, ME, USA). The mice were maintained under specific pathogen-free conditions in accordance with the ethical guidelines for the care of these mice at the Comparative Research Center of Rush University Medical Center. The mice were 6–8 weeks of age at the time of transplant.

For 1703 and 1703CPR cell transplantation, 10 million of disaggregated cells were resuspended in 100 of a 1:1 v/v mixture of cold DMEM:Matrigel (BD Biosciences, San Jose, CA) and kept on ice until transplantation. Cells were subcutaneously injected into the left flanks of NOD scid mice using 23 G needles. When tumors reached the size of 300 mm^3^, the mice were randomly divided into groups (6 mice/group) and treated with vehicle, CP (5 mg/kg, once), JIB04 (50 mg/kg/daily, 5 days/week), or CP plus JIB04. The mice were euthanized when the size of the tumors approached 2 ml in volume.

The lung adenocarcinoma PDX tumor (TM00199) was purchased from Jackson Laboratory. When the PDX tumor reached 1 cm^3^ volume, the tumor bearing mice were euthanized. Using aseptic technique, the tumor was removed and then minced into the smallest possible pieces using forceps and a scalpel. The minced tumors were transferred into a 1 ml syringe and then subcutaneously injected into 10 NSG mice (100 µl/mice) using a 14 G needle. When tumors reached 300 mm^3^, the mice were treated with CP (5 mg/kg, once). Tumor growth was then monitored with a caliper twice per week. Another round of CP was given after the tumors resumed growth. When tumors reached 1 cm^3^ volume, the tumors were transferred to another NSG mice with the same treatment repeated until the tumors became non-responsive.

### Statistical analysis

For in vitro experiments, one-way analysis of variance (ANOVA) and Student’s *t*-test were used to determine the statistical significance of differences among experimental groups. *P* values less than 0.05 are considered to be significantly different. For in vivo tumor growth, one-way ANOVA and Tukey post hoc test was used to determine the statistical significance between control and experimental groups.

## Supplementary Material

Supplemental Material
