# Supplementary material for: Inhibitors of Jumonji C domain-containing histone lysine demethylases overcome cisplatin and paclitaxel resistance in non-small cell lung cancer through APC/Cdh1-dependent degradation of CtIP and PAF15
Source: Cancer Biol Ther. 2022 Jan 31;23(1):65–75. doi: 10.1080/15384047.2021.2020060 (PMC8812751; doi:10.1080/15384047.2021.2020060)
Supplement: Supplemental Material [file KCBT_A_2020060_SM5347.zip › supplementary/sup data.pdf]

Supplemental figure legend

Fig. S1. Acquired resistance to CP and PTX correlates with increased expression of KDM genes. A. NSCLC cell lines A549, 1703, 1975 and their CP resistant derivatives (CPR) were treated with vehicle (NT) or CP (10 µM) for 24h. B. A549 and its PTX resistant derivatives were treated with vehicle (NT) or PTX (100 nM) for 24h. Expression of the indicated genes was determined by PCR Array. Relative gene levels are listed (red color indicates an increase while green color indicates a decrease). Note that KDM3A, KDM4B, KDM5A, KDM6A are commonly increased in all CP-resistant cell lines (cutoff: 1.5 fold increase). KDM4B, KDM5A, KDM6A are also increased in PTX resistant A549 cells.

Figure S2. HR inhibitor sensitizes resistant cells to CP. CPR cells were treated with CP (1 uM) +/- the HR inhibitor RI-1 for 48 hr. Colony formation was determined 10 days after drug removal. There are significant differences between RI-1 and RI-1+CP in A549CPR ($p=0.000$) and 1703CPR ($p=0.000$) cells.

Figure S1

A.

	A549		A549 CPR		1703		1703 CPR		1975		1975 CPR	
	NT	CP	NT	CP	NT	CP	NT	CP	NT	CP	NT	CP
KDM1A	1.00	1.56	0.85	4.84	1.00	0.52	0.70	1.03	1.00	1.22	1.14	1.44
KDM1B	1.00	2.01	1.55	2.03	1.00	0.47	0.30	0.80	1.00	1.00	1.10	1.03
KDM2A	1.00	2.08	1.10	4.13	1.00	1.05	1.10	2.13	1.00	1.12	0.76	0.92
KDM2B	1.00	0.25	0.21	0.33	1.00	0.73	0.85	1.69	1.00	1.02	0.79	0.71
KDM3A	1.00	2.04	1.59	8.43	1.00	1.09	1.71	3.33	1.00	1.23	1.57	1.95
KDM3B	1.00	0.31	0.21	0.34	1.00	0.35	0.51	0.76	1.00	1.02	0.86	0.85
KDM4A	1.00	1.26	0.92	0.47	1.00	0.94	1.11	1.23	1.00	1.01	1.66	1.71
KDM4B	1.00	2.15	1.32	3.08	1.00	0.96	1.78	1.96	1.00	0.96	1.52	2.20
KDM4C	1.00	0.97	0.97	0.72	1.00	0.56	0.54	0.43	1.00	0.98	1.17	1.46
KDM4D	1.00	2.01	3.51	3.20	1.00	0.61	1.56	2.63	1.00	0.76	0.80	0.89
KDM5A	1.00	0.96	3.74	4.52	1.00	0.78	1.16	3.58	1.00	0.83	0.84	2.10
KDM5B	1.00	0.95	0.77	1.27	1.00	0.70	0.97	1.59	1.00	0.93	0.93	0.85
KDM5C	1.00	1.67	1.71	1.75	1.00	0.93	1.22	3.18	1.00	0.96	1.04	1.10
KDM5D	1.00	0.90	0.97	1.02	1.00	0.46	0.87	1.10	1.00	0.61	0.73	1.47
KDM6A	1.00	2.40	2.49	6.27	1.00	1.14	1.69	3.06	1.00	1.16	1.39	1.87
KDM6B	1.00	1.22	0.60	1.23	1.00	0.45	0.44	1.00	1.00	1.18	0.92	0.95
KDM7A	1.00	0.70	0.90	2.56	1.00	0.82	1.68	2.48	1.00	1.11	1.02	1.09
KDM8	1.00	0.35	0.32	0.42	1.00	0.57	0.99	1.98	1.00	1.00	0.94	0.97
PHF8	1.00	0.79	0.52	0.78	1.00	0.53	0.78	1.84	1.00	0.79	0.52	0.78

B.

	A549		A549 PTXR	
	NT	PTX	NT	PTX
KDM1A	1.00	1.11	0.99	0.84
KDM1B	1.00	1.08	0.91	0.94
KDM2A	1.00	0.99	0.82	1.06
KDM2B	1.00	1.27	0.89	1.29
KDM3A	1.00	1.04	1.08	1.15
KDM3B	1.00	1.17	0.97	0.96
KDM4A	1.00	1.36	1.11	1.51
KDM4B	1.00	1.67	1.52	2.53
KDM4C	1.00	1.02	1.12	1.08
KDM4D	1.01	1.22	1.56	1.87
KDM5A	1.00	1.23	1.57	1.78
KDM5B	1.00	1.12	0.67	1.04
KDM5C	1.00	1.26	1.25	1.40
KDM5D	1.00	1.32	1.08	1.22
KDM6A	1.00	1.49	1.68	1.88
KDM6B	1.00	1.58	1.47	2.69
KDM7A	1.00	1.23	1.67	2.21
KDM8	1.00	1.20	1.58	2.18
PHF8	1.00	1.03	1.38	1.40

Figure S2

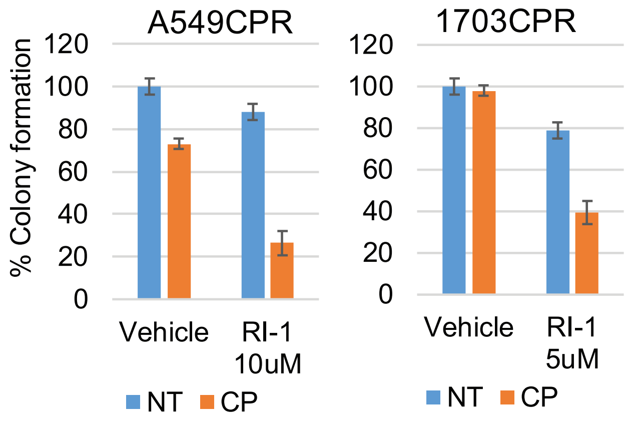
A549CPR
% Colony formation
120
100
80
60
40
20
0
Vehicle
RI-1 10uM
NT
CP
1703CPR
120
100
80
60
40
20
0
Vehicle
RI-1 5uM
NT
CP

Table S1. Primer sequences for KDMs

	FORWARD	REVERSE
KDM1A	TGACCGGATGACTTCTCAAGA	GTTGGAGAGTAGCCTCAAATGTC
KDM1B	CTCTCCTGTGGGGAACATTTC	GACTAGGTTCGGTTTTGCCATT
KDM2A	CCAAAGGTGCGGGTTCCTAC	GGCTCCTGACACAATCGGG
KDM2B	GGGTTCCCCTGATATTTCGAGA	GCTCCCCACTAGGAGTTTGAC
KDM3A	ACAGTGGCCTGCAATAACGTA	TCCCAGAAAGCGAACAGAAGT
KDM3B	GCTTCGGTTCCTGTCAGATG	GACCTCGGCTGAATATCTCTTG
KDM4A	ATCCCAGTGCTAGAGTAATGACC	ACTCTTTTGGAGGAACAACCTTG
KDM4B	ACTTCAACAAATACGTGGCCTAC	CGATGTCATACGTCTGCC
KDM4C	CGAGGTGGAAAGTCCTCTGAA	GGGCTCCTTTAGACTCCATGTAT
KDM4D	TAGAAGGCGTCAATACACCCT	GGGGCACCACATACCAAGTT
KDM5A	GTCTAAAGTGGGTAGTCGCTTG	GTTTGGGTATCAGTGCTGAGAA
KDM5B	AGTGGGCTCACATATCAGAGG	CAAACACCTTAGGCTGTCTCC
KDM5C	TCAGTGACAGTAAACGGCACC	ACACCGGCATCACATTTAGGT
KDM5D	TAACACACACCCGTTTGACAA	GCTGCTGAACTTTGAAGGCTG
KDM6A	TACAGGCTCAGTTGTGTAACCT	CTGCGGGAATTGGTAGGCTC
KDM6B	CGCTGCCTCACCCATATCC	ATCCGCGACCTCTGAACTCT
JHDM1D	GTGGAGGTCCCTGATATAGCC	CCACCGAAGTCAATGTGGAAA
KDM8	GGCCCGTGATCCTGAAAGG	ACCTCGAACCAACTTCCACTG
